# Effect of Health Intervention *via* Web-Based Education on Improving Information-Motivation-Behavioral Skills Related to HPV Vaccination Among Chinese Female College Students

**DOI:** 10.3389/ijph.2023.1605596

**Published:** 2023-02-03

**Authors:** Hong Wang, Xiaoyang Wang, Peipei Chen, Huifang Xu, Yin Liu, Ruihua Kang, Liyang Zheng, Xingyuan Sun, Xibin Sun, Shaokai Zhang

**Affiliations:** Department of Cancer Epidemiology, The Affiliated Cancer Hospital of Zhengzhou University & Henan Cancer Hospital, Zhengzhou, China

**Keywords:** human papillomavirus (HPV), HPV vaccines, health intervention, information-motivation-behavioral skills (IMB) model, China

## Abstract

**Objectives:** The human papillomavirus (HPV) vaccination rate is generally low in China. This study aimed to assess the effect of web-based education on improving information-motivation-behavior skills related to HPV vaccination among Chinese female college students.

**Methods:** From February to May 2020, female students were recruited from a university and divided into intervention and control groups. The intervention group received 7 days of HPV-related web-based education. Related information were collected using questionnaires in the baseline, 7 days, 1 month, and 3 months after the intervention. Chi-square test and repeated ANOVA were used to compare the differences between the two groups in the four surveys.

**Results:** A total of 449 students (235 in the intervention and 214 in the control group) were included in the analysis. There were no statistical differences in demographic information between the two groups. Compared with the control group, students in the intervention group showed a richer knowledge and subjective norms of HPV vaccination (*p* < 0.05).

**Conclusion:** The study provides preliminary support for a health intervention *via* web-based education in increasing HPV vaccination information among female college students.

## Introduction

Cervical cancer is the fourth most common malignancy among women worldwide, with 604,127 new cases and 341,831 deaths in 2020 [1]. As the most populous country, China accounts for 18.2% and 17.3% of global incidence and mortality, respectively [1]. According to the latest Chinese Cancer Registry Annual Report, there were approximately 111,000 new cases and 34,000 deaths in 2015, ranking 6th in incidence and 8th in mortality respectively among female malignant tumors [2]. The incidence of cervical cancer has increased in China over the past 20 years [3], which is a remarkable public health burden.

It has been well acknowledged that persistent infection of high-risk human papillomavirus (HPV) is the primary cause of cervical cancer [4]. Fortunately, prophylactic HPV vaccines are available and have been approved for the primary prevention of cervical cancer in over 100 countries. Given the availability of effective prevention measures for cervical cancer, the World Health Organization launched a global strategy to accelerate the elimination of cervical cancer as a public health problem, and the “90-70-90 targets” must be met by 2030 for countries: the first “90” means “90% of girls fully vaccinated with the HPV vaccine by 15 years of age” [5]. Therefore, increasing the coverage of prophylactic HPV vaccines is an essential part of the global effort to eliminate cervical cancer. As a critical part of achieving this global goal, the “Healthy China Action—Implementation Plan for Cancer Prevention and Control (2019-2022)”—has been developed by the Chinese government. The action plan proposed promoting HPV vaccination in the population, which provided a great opportunity to increase the HPV vaccination coverage [6]. However, imported HPV vaccines were not introduced in mainland China until 2016 [7], and before that, the awareness and acceptance of HPV vaccines were generally low [8–10]. This situation has improved since three imported HPV vaccines and one domestic vaccine were approved in China [11, 12]. Nevertheless, China still has a long way to go to improve the HPV vaccination coverage rate for accelerating the global elimination of cervical cancer [13].

Education plays a crucial role in HPV vaccine awareness and acceptance in the general population [14, 15]. Studies have demonstrated that an educational intervention may improve HPV-related knowledge and HPV vaccine acceptance [16–18]. However, at present, domestic health education is mostly based on communities, schools, hospitals, and other offline places. This traditional education requires enormous human and material resources, and it is not easy to disseminate HPV-related knowledge among the vast population in China. Therefore, there are no quick and effective strategies to improve HPV vaccination coverage in China so far. Currently, with the internet being a necessity in daily life, most young people tend to use the internet to get health information [19]. Compared with traditional education, web-based education is flexible and diverse, instant, and fast. Moreover, the content can be customized to meet the needs of different regions, nationalities, and cultural backgrounds. In the current situation of limited medical staff, web-based education would be a cost-effective method to disseminate HPV-related knowledge. Furthermore, the information-motivation-behavioral skills (IMB) model is a well-validated approach for the prediction and promotion of health behavior performance [20]. According to the theory, a person with rich knowledge will have the intention to practice healthy behaviors when he/she has motivation, the ability/skills to complete healthy behaviors, and self-efficacy, and the intention will be easily transformed into actual practice when objective conditions permit. Based on the theory of IMB model, we designed health education materials for HPV vaccine, including HPV knowledge, vaccination motivation and behavioral skills, and conducted a web-based education intervention study for female college students who are at high risk of HPV infection. This study evaluated the intervention effect of the web-based education on female college students. And the results are expected to provide a reference for improving the HPV vaccination rate.

## Methods

### Study Design and Population

A school-based, prospective, intervention study was conducted involving a 7-day web-based education intervention with a further 3-month follow-up. From February 2020 to May 2020, female first-year students were recruited online from a university in Zhengzhou, Henan Province through convenience sampling. An electronic version of the informed consent form was sent to voluntary participants. The inclusion criteria are as follows: 1) females 18 years of age and more; 2) first-year undergraduates; 3) no vaccination contraindications. The exclusion criteria are as follows: 1) males; 2) females under 18; 3) non-undergraduate first-year students; 4) medical students; 5) previous history of vaccination contraindications; 6) current pregnancy or breast feeding.

Ethical approval for the study was obtained from the Institutional Review Board of the Chinese Center for Disease Control and Prevention on 24 October 2019. Before enrolment in the study, participants were well informed of the purpose, methods, expected risks, and benefits of this research. All participants voluntarily signed informed consent.

### Intervention and Measurements

The participants were divided on a class level to intervention and control groups after stratification by liberal arts and sciences, and were invited to join the corresponding DingTalk team. DingTalk is a free mobile office platform for communication and collaboration created by Alibaba. The students in the intervention group received 7 days of HPV-related web-based education, while the control group received just popular science education (not HPV-related). To maintain students’ interest in the control group, the non-HPV publicity materials were uploaded at the same time every day. The content of the HPV-related web-based education was developed under the guidance of IMB model after reviewing the related literature and conducting several rounds of panel discussions. It contains 2-day materials to popularize HPV knowledge, 2-day materials on situational stories to motivate participants to vaccinate themselves against HPV and 3-day materials on objective skills with self-decision making, self-efficacy, and objective conditions for making an appointment and receiving the HPV vaccine. The above intervention materials were designed as online readable texts or videos that could be easily accessed by the target population, see Supplementary File S1 for details. The education was delivered daily for seven consecutive days, 10 minutes per day.

Information from participants was collected using a structured electronic questionnaire four times: at the study enrollment, after completing the 7 days of web-based education intervention, 1 month, and 3 months after the intervention. The content of the questionnaire includes sociodemographic information, participants’ information concerning HPV, motivation for vaccination, and behavioral skills regarding HPV vaccination.

For the information concerning HPV, there were 11 items, and the answers were “Agree,” “Disagree,” or “I do not know” [21, 22]. Each correct answer scored 1 point, while incorrect or unknown answers scored 0 points. The total score of the information questions was derived by adding the number of correct responses. Motivation for vaccination was measured in 5 dimensions with a total of 19 items: perceived susceptibility (2 items), perceived severity (4 items), perceived benefits (3 items) and barriers (5 items), and subjective norms (5 items) [22–25]. Behavioral skills were evaluated in 2 dimensions with a total of 11 items, including perceived control in decision-making about HPV vaccination (3 items), and perceived self-efficacy (8 items) [20, 21, 25]. The answers to these items were measured on a 5-point Likert scale (1 = strongly disagree, 2 = disagree, 3 = neither disagree nor agree, 4 = agree, 5 = strongly agree). The measurement items can be found in Supplementary File S2. The total score of each dimension was obtained by summing the responses of the items in the corresponding dimension. Except for the perceived barriers, the higher scores on other items indicated that the participants were more likely to receive the HPV vaccine. The Cronbach’s alpha of information, motivation, and behavioral skills were 0.740, 0.726, and 0.855, respectively, indicating that the three latent variables had adequate internal consistency. The Kaiser-Meyer-Olkin Measure of Sampling Adequacy (KMO) value was 0.812, and Bartlett’s Test of Sphericity (BTS) value was statistically significant (*p* < 0.001).

### Data Collection and Quality Control

The online questionnaire was delivered through the DingTalk team. Participants in the corresponding team could complete the questionnaire anonymously and independently using DingTalk. The content of the questionnaire did not involve any personal information related to the participants. After the questionnaire was completed, the trained investigators checked and corrected the questionnaire logically.

### Statistical Analysis

Chi-square or Fisher’s exact test analysis was used to compare demographic information, and information concerning HPV at baseline and 3 months after intervention between the intervention, and control groups. Since the information, motivation and behavioral skills of each participant were repeatedly measured four times, ANOVA of repeated measurement data was used to evaluate the effect, and the resulting variables of subjects who lost the follow-up were analyzed according to the missing values. Statistical analysis was performed using SAS 9.4. Statistical significance was set at *p* < 0.05 (2-tailed test).

## Results

### Baseline Characteristics of Students

**FIGURE 1:**
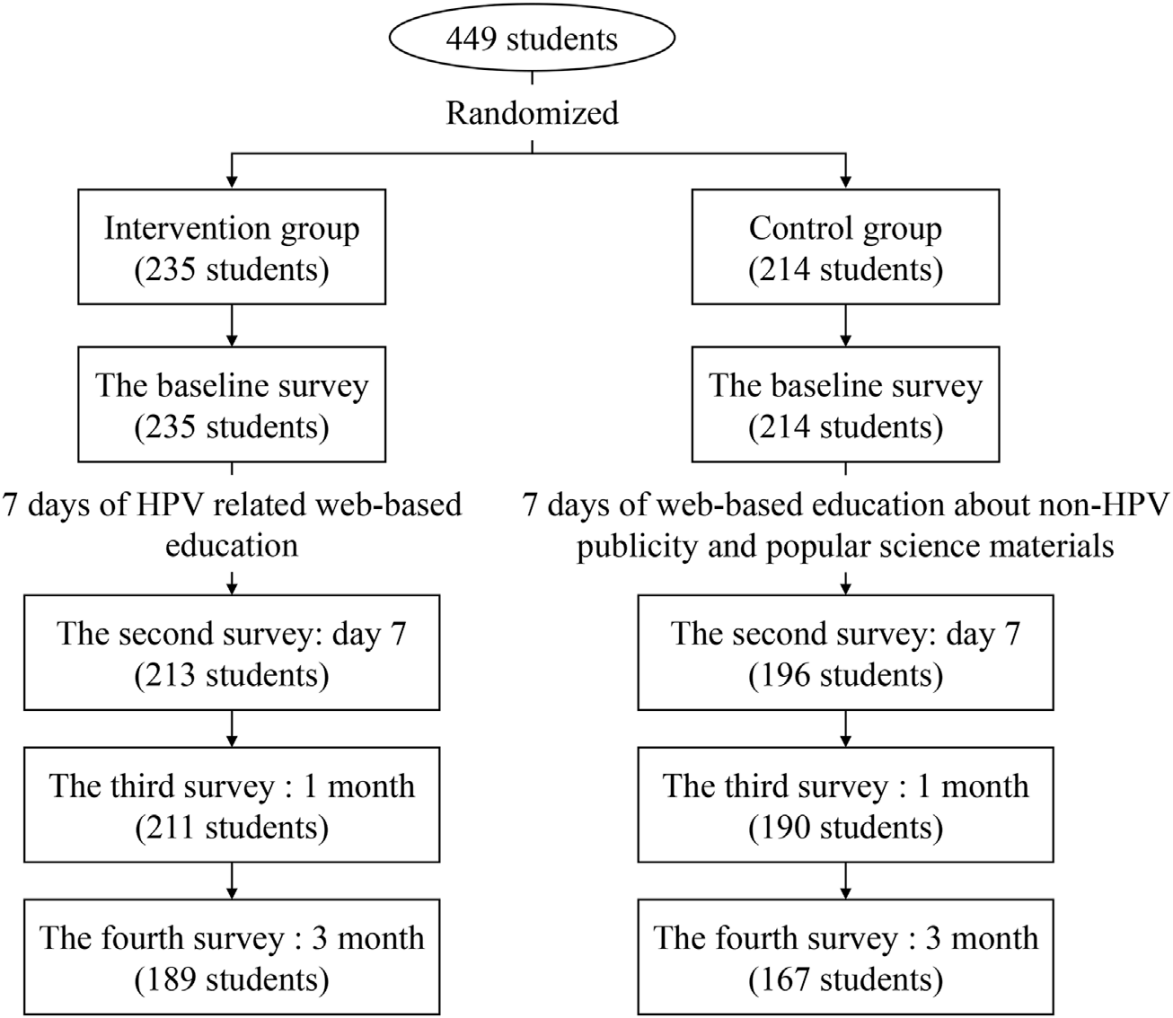
Flow chart of the study (China. 2020).

455 students were recruited to the project, of which 6 students (1.3%) had HPV vaccination, and the remaining 449 unvaccinated students were included in the final analysis. In the baseline, there were 235 students in the intervention group and 214 students in the control group. In the follow-up survey, 93 students were lost, and only 356 students completed the fourth survey, including 189 in the intervention group and 167 in the control group (Figure 1).

Most students were Han Ethnicity (98.9%) and lived in rural areas (56.6%). 63.0% of students had an average living expense of 1,000 to 2,000 Chinese Yuan (CNY) per month. Among the subjects, 77.1% of their parents were in the state of marriage, and 60.1% of them had junior high school education or below. About 52.6% and 51.0% of the students had heard of HPV-related diseases and the HPV vaccine, respectively. There were no statistical differences in demographic information between the intervention and control groups. More details are shown in Table 1.

**TABLE 1:** Baseline characteristics of subjects (China, 2020).

Variables	All N (%)	Intervention group n (%)	Control group n (%)	*p*
Ethnicity				0.673
Han	444 (98.9)	233 (99.2)	211 (98.6)	
Other	5 (1.1)	2 (0.8)	3 (1.4)	
Permanent residence (for more than 1 year)				0.058
Urban	195 (43.4)	112 (47.7)	83 (38.8)	
Rural	254 (56.6)	123 (52.3)	131 (61.2)	
Marital status of parents				0.638
In marriage	346 (77.1)	179 (76.2)	167 (78.0)	
Other	103 (22.9)	56 (23.8)	47 (22.0)	
Education level of parents				0.570
Junior high school or below	270 (60.1)	136 (57.9)	134 (62.6)	
Senior high school (including vocational high School)	137 (30.5)	75 (31.9)	62 (29.0)	
College (including technical college) and above	42 (9.4)	24 (10.2)	18 (8.4)	
Living Expenses (RMB/month)				0.292
<1000 yuan	154 (34.3)	86 (36.6)	68 (31.8)	
1000-2000 yuan	283 (63.0)	141 (60.0)	142 (66.3)	
>2000 yuan	12 (2.7)	8 (3.4)	4 (1.9)	
Major in school				0.651
Science	209 (46.5)	107 (45.5)	102 (47.7)	
Liberal art	240 (53.5)	128 (54.5)	112 (52.3)	
Ever heared of HPV				0.640
Yes	234 (52.1)	120 (51.1)	114 (53.3)	
No	215 (47.9)	115 (48.9)	100 (46.7)	
Ever heard of HPV-related diseases				0.779
Yes	236 (52.6)	125 (53.2)	111 (51.9)	
No	213 (47.4)	110 (45.8)	103 (48.1)	
Ever heard of the HPV vaccine				0.685
Yes	229 (51.0)	122 (51.9)	107 (50.0)	
No	220 (49.0)	113 (48.1)	107 (50.0)	

CNY, Chinese Yuan; HPV, human papillomavirus.

### HPV-Related Knowledge

#### Pre-Intervention HPV-Related Information

There were 11 items about HPV-related information. At baseline, only 5 items had more than 50% accuracy. About 70% of the students knew that “HPV is related to the development of cervical cancer,” and more than 70% knew that “Regular cervical cancer screening is necessary after HPV vaccination” and that “The HPV vaccine does not protect against all types of cervical cancer.” More than 50% of the students knew that “HPV infection may result in oral cancer, condyloma acuminatum, and anal cancer.” Two items had less than 10% accuracy, including “HPV is almost asymptomatic” and “Most HPV infections will disappear on their own.” There were no statistical differences in all the 11 items between the intervention and control groups. More details are shown in Table 2.

**TABLE 2:** HPV-related information among subjects in the baseline and 3 months after the intervention (China, 2020).

Variables	Baseline			3 months		
	Intervention group n (%)	Control group n (%)	*p*	Intervention group n (%)	Control group n (%)	*p*
HPV is related to the development of cervical cancer			0.448			<0.001
Agree^a^	168 (71.5)	145 (67.8)		174 (92.1)	137 (82.0)	
Disagree	8 (3.4)	5 (2.3)		7 (3.7)	3 (1.8)	
Do not know	59 (25.1)	64 (29.9)		8 (4.2)	27 (16.2)	
Males cannot be infected with HPV			0.120			<0.001
Agree	19 (8.1)	24 (11.2)		13 (6.9)	14 (8.4)	
Disagree^a^	143 (60.8)	110 (51.4)		162 (85.7)	109 (65.3)	
Do not know	73 (31.1)	80 (37.4)		14 (7.4)	44 (26.3)	
HPV is related to sexual behavior			0.617			<0.001
Agree^a^	199 (42.1)	98 (45.8)		146 (77.2)	102 (61.1)	
Disagree	23 (9.8)	23 (10.7)		23 (12.2)	21 (12.6)	
Do not know	113 (48.1)	93 (43.5)		20 (10.6)	44 (26.3)	
Condoms can prevent HPV infection			0.675			0.002
Agree^a^	53 (22.6)	50 (23.4)		75 (39.7)	61 (36.5)	
Disagree	68 (28.9)	54 (25.2)		69 (36.5)	39 (23.4)	
Do not know	114 (48.5)	110 (51.4)		45 (23.8)	67 (40.1)	
HPV is almost asymptomatic			0.575			0.086
Agree^a^	15 (6.4)	9 (4.2)		27 (14.3)	28 (16.8)	
Disagree	142 (60.4)	130 (60.7)		139 (73.5)	106 (63.5)	
Do not know	78 (33.2)	75 (35.1)		23 (12.2)	33 (19.8)	
The HPV vaccine protects against all types of cervical cancer			0.839			0.005
Agree	10 (4.3)	8 (3.7)		13 (6.9)	16 (9.6)	
Disagree^a^	172 (73.2)	153 (71.5)		161 (85.2)	120 (71.9)	
Do not know	53 (22.5)	53 (24.8)		15 (7.9)	31 (18.6)	
HPV infection may result in oral cancer, condyloma acuminatum, and anal cancer			0.268			<0.001
Agree^a^	133 (56.6)	110 (51.4)		164 (86.8)	116 (69.5)	
Disagree	20 (8.5)	14 (6.5)		12 (6.3)	12 (7.2)	
Do not know	82 (34.9)	90 (42.1)		13 (6.9)	39 (23.3)	
Most HPV infections will disappear on their own			0.988			<0.001
Agree^a^	15 (6.4)	14 (6.5)		35 (18.5)	23 (13.8)	
Disagree	106 (45.1)	95 (44.4)		131 (69.3)	89 (53.3)	
Do not know	114 (48.5)	105 (49.1)		23 (12.2)	55 (32.9)	
HPV infection is very common			0.116			0.002
Agree^a^	57 (24.3)	45 (21.0)		112 (59.2)	70 (41.9)	
Disagree	70 (29.8)	50 (23.4)		37 (19.6)	36 (21.6)	
Do not know	108 (45.9)	119 (55.6)		40 (21.2)	61 (36.5)	
Regular cervical cancer screening is unnecessary after HPV vaccination			0.532			0.004
Agree	1 (0.4)	3 (1.4)		7 (3.7)	2 (1.2)	
Disagree^a^	173 (73.6)	158 (73.8)		168 (88.9)	135 (80.8)	
Do not know	61 (26.0)	53 (24.8)		14 (7.4)	30 (18.0)	
The best time for HPV vaccination is before any experience of sexual contact			0.213			<0.001
Agree^a^	79 (33.6)	68 (31.8)		166 (87.8)	108 (64.7)	
Disagree	17 (7.2)	8 (3.7)		7 (3.7)	4 (2.4)	
Do not know	139 (59.2)	138 (64.5)		16 (8.5)	55 (32.9)	

^a^ Appropriate response; HPV, human papillomavirus.

#### Post-Intervention HPV-Related Information

Three months after the intervention, the accuracy improved in both groups, and there were 7 items with more than 50% accuracy. Except for the item, “HPV is almost asymptomatic,” the accuracy of the other items in the intervention group was higher than in the control group (all *p* < 0.05). More details are shown in Table 2.

### Changes in Information-Motivation-Behavioral Skills Related to HPV Vaccination

**FIGURE 2:**
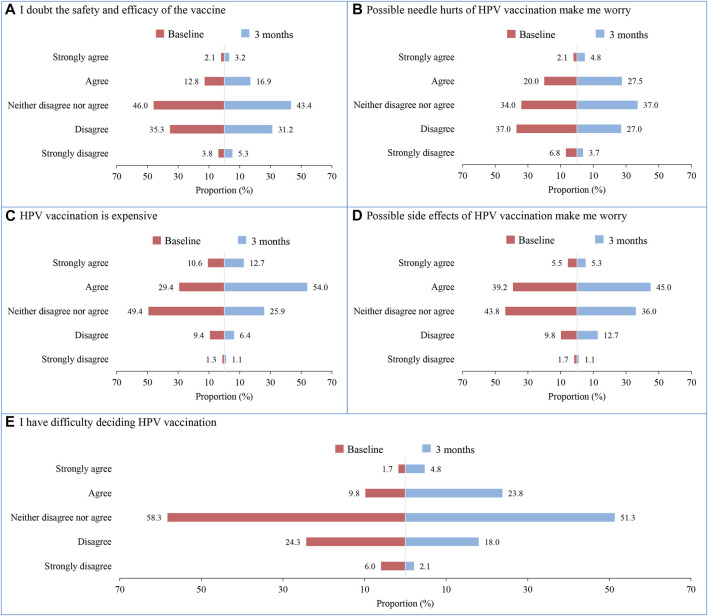
Changes of perceived barriers of human papillomavirus vaccination among students in the intervention group before and after intervention. **(A)** I doubt the safety and efficacy of the vaccine; **(B)** Possible needle hurts of HPV vaccination make me worry; **(C)** HPV vaccination is expensive; **(D)** Possible side effects of HPV vaccination make me worry; **(E)** I have difficulty deciding HPV vaccination (China. 2020).

Compared with the baseline, the mean score of “information,” “perceived susceptibility,” “subjective norms,” and “self-efficacy” increased significantly within 3 months after the intervention (all *p* < 0.05). Statistically significant differences were observed between the groups in the scores of “information” and “subjective norms,” with higher scores in the intervention group (*p* < 0.05). Notably, there was an increase in the score of “perceived barriers” but a decrease in the score of “decision-making” in both groups within 3 months of intervention (*p* < 0.05). See Table 3 for more details. Taking perceived barriers as an example, compared with the baseline, the proportion of “Agree” or “Strongly agree” in the 5 items increased 3 months after the intervention, of which “HPV vaccination is expensive” increased the most (Figure 2).

**TABLE 3:** Changes in the dimensions of the information-motivation-behavioral skills (IMB) model in the intervention and control groups (China, 2020).

Dimensions of the IMB model	Group	Score				*p*		
		Baseline	7 days	1 month	3 months	Group	Time	Group*Time
Information	Intervention	4.71 ± 2.58	7.34 ± 1.81	7.40 ± 1.97	7.35 ± 1.90	<0.001	<0.001	<0.001
	Control	4.49 ± 2.48	5.48 ± 2.69	5.68 ± 2.84	6.04 ± 2.73		<0.001	
Motivation								
Perceived susceptibility	Intervention	4.52 ± 1.75	5.36 ± 1.91	5.29 ± 1.88	5.25 ± 1.95	0.356	<0.001	0.012
	Control	4.79 ± 1.75	5.19 ± 1.58	5.21 ± 1.57	5.43 ± 1.78		<0.001	
Perceived severity	Intervention	13.70 ± 3.00	13.69 ± 3.03	13.49 ± 2.93	13.43 ± 3.31	0.926	0.877	0.014
	Control	14.01 ± 3.01	13.31 ± 2.81	13.12 ± 2.88	13.49 ± 3.01		<0.001	
Perceived benefits	Intervention	11.73 ± 1.73	11.89 ± 1.77	11.87 ± 1.72	11.79 ± 1.89	0.077	0.649	0.012
	Control	11.65 ± 1.70	11.37 ± 1.56	11.28 ± 1.43	11.69 ± 1.36		0.005	
Perceived barriers	Intervention	15.00 ± 2.62	15.58 ± 2.77	15.55 ± 2.94	16.07 ± 3.17	0.127	<0.001	0.292
	Control	15.26 ± 3.06	16.00 ± 2.81	16.27 ± 2.69	16.24 ± 2.84		<0.001	
Subjective norms	Intervention	15.69 ± 2.26	15.76 ± 2.70	15.78 ± 2.76	16.20 ± 2.78	0.018	<0.001	0.055
	Control	15.18 ± 2.40	15.11 ± 2.19	14.93 ± 2.40	15.49 ± 2.87		0.013	
Behavioral skills								
Decision-making	Intervention	12.17 ± 1.40	11.53 ± 1.46	11.57 ± 1.38	11.50 ± 1.40	0.524	<0.001	0.658
	Control	11.99 ± 1.49	11.47 ± 1.56	11.38 ± 1.28	11.41 ± 1.36		<0.001	
Self-efficacy	Intervention	26.75 ± 4.12	27.05 ± 4.34	27.09 ± 4.50	27.33 ± 4.53	0.701	0.043	0.732
	Control	26.41 ± 4.33	26.40 ± 4.55	26.44 ± 4.36	27.06 ± 4.50		0.273	

IMB, information-motivation-behavioral skills.

## Discussion

This study explored the effect of web-based education on improving college female students’ information-motivation-behavior skills related to HPV vaccination. The results showed that web-based education could significantly improve the HPV-related information in female college students. However, the effect on motivation and behavior skills related to HPV vaccination may require a longer follow-up. The results may provide a reference for the future promotion of HPV vaccination.

The results of our study showed that Chinese female college students had a low understanding of HPV, indicating that although it has been 4 years since the HPV vaccine was approved in China [7], the awareness of HPV still needs to be further improved. It should be also noted that the college students’ awareness of HPV is not high, indicating other populations may be lower, reflecting the urgent need for health education. Studies have shown that traditional health education could improve students’ knowledge of HPV within a short time [26, 27]. As a new form of education, web-based education has many advantages over traditional education, which can maximize the utilization of resources and improve the educational accessibility of people in different regions. In addition, since HPV is related to sexual behavior, web-based education can also protect privacy, avoid embarrassment, and improve participation. Based on an intervention study, our study showed that after a 7-day’s web-based education, the information related to HPV vaccination among female college students was significantly improved and lasted for at least 3 months, offering preliminary support for the importance of the health intervention *via* web-based education in increasing HPV vaccination information among female college students.

It is worth noting that after 7 days of web-based education, there were increases in students’ barriers to HPV vaccination, such as more doubts about the safety and effectiveness of the vaccine, more concerns about the side effects and pain of the vaccine. It is speculated that the reason may be that students pay more attention to the safety and effectiveness of vaccine after they have a certain understanding of it. So far, many studies have proven the safety and efficacy of the HPV vaccine [28–30], therefore, further education should enhance the safety, efficacy, and adverse events of HPV vaccines to eliminate the doubts. In addition to the worry about the safety and effectiveness, the high price of the vaccines makes it difficult for students to accept. At present, there were three imported vaccines available in China such as the bivalent HPV (Cervarix, $262 for three doses), quadrivalent HPV (Gardasil, $360 for three doses), and non-avalent HPV (Gardasil 9, $586 for three doses) [7], all these vaccines are costly and makes it not affordable for low to middle-income families. Fortunately, the Chinese Food and Drug Administration licensed the first Chinese domestic HPV vaccine against HPV 16 and 18 (Cecolin, Innovax, Xiamen, China) on Dec 31, 2019, which is priced at ¥329 ($47.7) per dose (about half of the price of Cervarix) and has similar efficacy to Cervarix [7, 28, 31]. The reduction in the cost of vaccination with Cecolin raises hope for the wide promotion of HPV vaccine.

The study found a significant increase in HPV-related knowledge through web-based education, which was consistent with other study [32]. However, the improvement in motivation and behavioral skills for HPV vaccination was limited, suggesting that further studies with a more extended follow-up period are needed to investigate the effect. According to the IMB model, HPV vaccine uptake motivation was mainly affected by individuals’ attitudes towards HPV vaccination as well as perceptions of social support for undergoing HPV vaccination [20]. Attitudes towards HPV vaccination are based upon the individual’s values about the vaccination outcomes and evaluations of these outcomes. Perceptions of social support for HPV vaccination are a function of perceived support from significant others, such as parents, partners, or healthcare providers, and the individual’s motivation to comply with these significant others’ wishes. Studies found a relationship between perceptions of social support from significant others for vaccine uptake and vaccination status [20, 33]. Therefore, it is speculated that the limited improvement of motivation related to HPV vaccination may in part be due to the low acceptance of the HPV vaccine by significant others. On the other hand, social factors may indirectly impact motivation or behavioral skills. These factors include a lack of insurance coverage and the existence of media or professional vaccine skepticism. In addition, the improvement of motivation and behavioral skills in HPV vaccination through health education is a continuous process, and the effects may not be observed in a 7-day web-based education. Long-term studies may be needed to see the effects of health education on motivation, behavioral skills, even vaccination willingness, and vaccination rates.

The results showed that students in the control group experienced increased HPV vaccination knowledge and perceived susceptibility but more perceived barriers and decreased decision-making in the post-intervention period. The reasons may be as follows: on the one hand, this study is not blind to the participants. Since the participants are students from the same school, they might discuss the contents of the survey with each other, making students in the control group also obtain some knowledge of HPV. On the other hand, the questionnaire in this study involved many HPV-related questions, and participants were surveyed repeatedly using this questionnaire four times. Thus, the students in the control group may inquire about HPV-related knowledge out of interest after finishing the questionnaire, which could improve their understanding of HPV-related information. However, such information is likely to be incomplete or negative, leading to a worse perception of HPV vaccination and decreased decision-making ability.

There are several limitations of the study: first, the participants in our study were not blinded, and contamination may occur. Second, the study was carried out among female college students in a university in Zhengzhou, Henan Province. The HPV knowledge and vaccine receptivity may vary widely based on region, sociodemographic factors, and education level, so the findings may not be generalized to the entire Chinese female population. Third, the HPV-related educational intervention only lasted for 3 months, which rendered its impact on motivation, behavioral skills, and vaccination rate not obvious.

In conclusion, our study found that web-based education could significantly improve the HPV-related information in female college students, but the effect on motivation and behavior skills related to HPV vaccination may require longer follow-up to be observed. Follow-up studies are needed to observe if an increased HPV-related knowledge might be translated into higher vaccination rates in China. The optimization of vaccination will need to be further enhanced to address the relevant barriers. The findings of our study could provide valuable information for future HPV vaccination policies during this critical time of the global acceleration towards the elimination of cervical cancer.
