# Supplementary material for: Effect of Health Intervention via Web-Based Education on Improving Information-Motivation-Behavioral Skills Related to HPV Vaccination Among Chinese Female College Students
Source: Int J Public Health. 2023 Feb 3;68:1605596. doi: 10.3389/ijph.2023.1605596 (PMC9935583; doi:10.3389/ijph.2023.1605596)
Supplement: Supplementary file 1 [file DataSheet2.docx]

**Supplementary File 2: The survey items of information, motivation, and behavioral skills regarding HPV vaccination** **(China, 2020)**

**1. HPV-related information**

Please judge whether the following statements are true or false

|  | True | False | Don’t know |
| --- | --- | --- | --- |
| HPV is related to the development of cervical cancer | □ | □ | □ |
| Males cannot be infected with HPV | □ | □ | □ |
| HPV is related to sexual behavior | □ | □ | □ |
| Condoms can prevent HPV infection | □ | □ | □ |
| HPV is almost asymptomatic | □ | □ | □ |
| The HPV vaccine protects against all types of cervical cancer | □ | □ | □ |
| HPV infection may result in oral cancer, condyloma acuminatum, and anal cancer | □ | □ | □ |
| Most HPV infections will disappear on their own | □ | □ | □ |
| HPV infection is very common | □ | □ | □ |
| Regular cervical cancer screening is unnecessary after HPV vaccination | □ | □ | □ |
| The best time for HPV vaccination is before any experience of sexual contact | □ | □ | □ |

**2. Motivation for HPV vaccination**

In daily life, everyone has their own understanding of "health". What is your attitude towards the following statements about HPV? (Please tick "√" in the option)

|  | [Strongly disagree](C:/Users/Administrator/AppData/Local/youdao/dict/Application/8.9.3.0/resultui/html/index.html#/javascript:;) | Disagree | Not sure | Agree | Strongly agree |
| --- | --- | --- | --- | --- | --- |
| **Perceived susceptibility** |  |  |  |  |  |
| I'm at risk for HPV. | □ | □ | □ | □ | □ |
| I'm at risk for cervical cancer. | □ | □ | □ | □ | □ |
| **Perceived severity** |  |  |  |  |  |
| Once infected with HPV, I’ll be very scared. | □ | □ | □ | □ | □ |
| HPV infection is very serious and will affect my campus life. | □ | □ | □ | □ | □ |
| Cervical cancer is devastating for me. | □ | □ | □ | □ | □ |
| Having cervical cancer will have a major impact on my life. | □ | □ | □ | □ | □ |
| **Perceived benefits** |  |  |  |  |  |
| You should get the HPV vaccine even if you have had sex. | □ | □ | □ | □ | □ |
| HPV vaccination helped protect me from HPV infection. | □ | □ | □ | □ | □ |
| HPV vaccination can reduce my risk of cervical cancer. | □ | □ | □ | □ | □ |
| **Perceived barriers** |  |  |  |  |  |
| I doubt the safety and effectiveness of the vaccine. | □ | □ | □ | □ | □ |
| It was difficult for me to decide to vaccinate against HPV. | □ | □ | □ | □ | □ |
| I'm worried about the sting of the HPV vaccine. | □ | □ | □ | □ | □ |
| HPV vaccines are expensive. | □ | □ | □ | □ | □ |
| I’m concerned the possible side effects of HPV vaccination. | □ | □ | □ | □ | □ |
| **Subjective norms** |  |  |  |  |  |
| Other girls of the same age are considering taking HPV vaccine. | □ | □ | □ | □ | □ |
| My family thinks I should get the HPV vaccine. | □ | □ | □ | □ | □ |
| I do what my family thinks I should do. | □ | □ | □ | □ | □ |
| My friend thinks I should get the HPV vaccine. | □ | □ | □ | □ | □ |
| I do what my friends think I should do. | □ | □ | □ | □ | □ |

**3. Behavioral Skills**

What is your attitude towards the following statements about HPV? (Please tick "√" in the option)

|  | [Strongly disagree](C:/Users/Administrator/AppData/Local/youdao/dict/Application/8.9.3.0/resultui/html/index.html#/javascript:;) | Disagree | Not sure | Agree | Strongly agree |
| --- | --- | --- | --- | --- | --- |
| **Self-decision making** |  |  |  |  |  |
| I can decide for myself whether or not to get the HPV vaccine. | □ | □ | □ | □ | □ |
| I take my health very seriously. | □ | □ | □ | □ | □ |
| It is important for me to prevent diseases and infections. | □ | □ | □ | □ | □ |
| **Self-efficacy** |  |  |  |  |  |
| Although HPV vaccines are expensive, I still believe that I can complete the HPV vaccination. | □ | □ | □ | □ | □ |
| Although it's a little painful, I still believe that I can complete the HPV vaccination. | □ | □ | □ | □ | □ |
| Although I'm a little worried about the possible side effects of vaccination，I still believe that I can complete the HPV vaccination. | □ | □ | □ | □ | □ |
| Although I need to get three doses of the vaccine，I still believe that I can complete the HPV vaccination. | □ | □ | □ | □ | □ |
| I can freely discuss with my parents/ guardians/ nurses/ doctors about whether to get the HPV vaccine. | □ | □ | □ | □ | □ |
| I know the location of vaccination, or I will find out the location through online search or telephone consultation. | □ | □ | □ | □ | □ |
| I know that the HPV vaccine is a self-funded vaccine and the price is affordable for me. | □ | □ | □ | □ | □ |
| If I want to get vaccinated, I will complete 3 shots by setting a reminder calendar, etc. | □ | □ | □ | □ | □ |
