# Supplementary material for: Effect of Health Intervention via Web-Based Education on Improving Information-Motivation-Behavioral Skills Related to HPV Vaccination Among Chinese Female College Students
Source: Int J Public Health. 2023 Feb 3;68:1605596. doi: 10.3389/ijph.2023.1605596 (PMC9935583; doi:10.3389/ijph.2023.1605596)
Supplement: Supplementary file 2 [file DataSheet1.docx]

**Supplementary File 1. Cover pages and contents of the web-based education (China, 2020).**

| Serial number | Cover page | Content |
| --- | --- | --- |
| 1 | 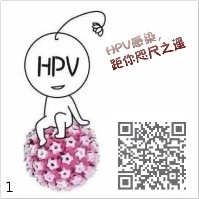 | General facts about HPV, including HPV infection and related diseases. For example, there are more than a hundred different types of HPV, some of which are high risk and linked to the development of cancers, including cervical cancers. |
| 2 | 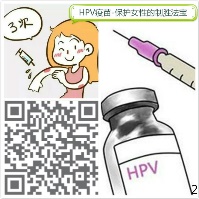 | Frequently asked questions and answers about HPV vaccine. For example, how does the HPV vaccine work? What are the recommended ages and populations for vaccination? |
| 3 | 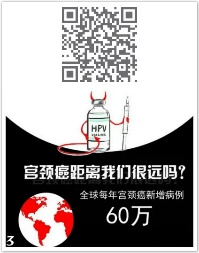 | A story of a female movie star who died of cervical cancer. Then, a case of a woman who missed HPV vaccination and cervical cancer screening, reached the terminal stage of cancer right after being found symptomatic, and died after painful treatment. Finally, the relevant facts about the screening, treatment, and prognosis of cervical cancer. |
| 4 | 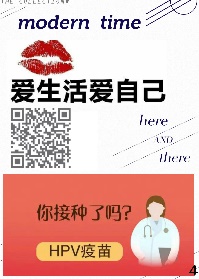 | Risk factors and early symptoms of cervical cancer, and ways to prevent and control cervical cancer. |
| 5 | 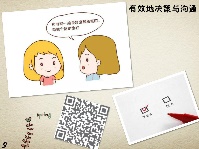 | The concept and practice of decision-making and effective communication. |
| 6 | 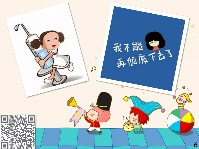 | The concept and function of self-efficacy, evaluation of self-efficacy, and ways to improve self-efficacy. |
| 7 | 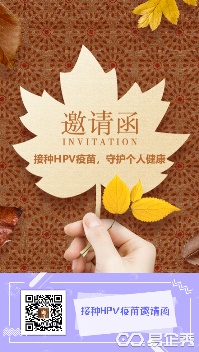 | Provide participants with the objective skills needed for HPV vaccination in the form of invitation letters designed with HTML5 front-end technology, such as appointment platform, price, vaccination venue, etc. |

HPV, human papillomavirus.
